# Performance of a Predictive Model for Long-Term Hemoglobin Response to Darbepoetin and Iron Administration in a Large Cohort of Hemodialysis Patients

**DOI:** 10.1371/journal.pone.0148938

**Published:** 2016-03-03

**Authors:** Carlo Barbieri, Elena Bolzoni, Flavio Mari, Isabella Cattinelli, Francesco Bellocchio, José D. Martin, Claudia Amato, Andrea Stopper, Emanuele Gatti, Iain C. Macdougall, Stefano Stuard, Bernard Canaud

**Affiliations:** 1 Fresenius Medical Care, Bad Homburg, Germany; 2 Intelligent Data Analysis Laboratory, University of Valencia, Burjassot (Valencia), Spain; 3 Center for Biomedical Technology at the Danube University, Krems, Austria; 4 Renal Unit, King's College Hospital, London, United Kingdom; 5 UFR Medicine, University of Montpellier I, Montpellier, France; University of Florida, UNITED STATES

## Abstract

Anemia management, based on erythropoiesis stimulating agents (ESA) and iron supplementation, has become an increasingly challenging problem in hemodialysis patients. Maintaining hemodialysis patients within narrow hemoglobin targets, preventing cycling outside target, and reducing ESA dosing to prevent adverse outcomes requires considerable attention from caregivers. Anticipation of the long-term response (i.e. at 3 months) to the ESA/iron therapy would be of fundamental importance for planning a successful treatment strategy. To this end, we developed a predictive model designed to support decision-making regarding anemia management in hemodialysis (HD) patients treated in center. An Artificial Neural Network (ANN) algorithm for predicting hemoglobin concentrations three months into the future was developed and evaluated in a retrospective study on a sample population of 1558 HD patients treated with intravenous (IV) darbepoetin alfa, and IV iron (sucrose or gluconate). Model inputs were the last 90 days of patients’ medical history and the subsequent 90 days of darbepoetin/iron prescription. Our model was able to predict individual variation of hemoglobin concentration 3 months in the future with a Mean Absolute Error (MAE) of 0.75 g/dL. Error analysis showed a narrow Gaussian distribution centered in 0 g/dL; a root cause analysis identified intercurrent and/or unpredictable events associated with hospitalization, blood transfusion, and laboratory error or misreported hemoglobin values as the main reasons for large discrepancy between predicted versus observed hemoglobin values. Our ANN predictive model offers a simple and reliable tool applicable in daily clinical practice for predicting the long-term response to ESA/iron therapy of HD patients.

## Introduction

In normal people, kidneys produce the hormone erythropoietin (EPO) in response to hypoxia; then, EPO stimulates the bone marrow (EPO target organ), to generate new blood cells. In chronic kidney disease (CKD) patients, as the degenerative and fibrosis process progresses, erythropoietin production is reduced and secondary anemia ensues. Besides low or inadequate circulating levels of erythropoietin, other conditions associated to chronic disease stage and to dialysis treatment (where present), such as uremic toxicity, iron deficiency, inflammation, malnutrition, or increased bleeding events exacerbate the level of anemic in these patients. Correction of anemia in dialysis patients represents a major target of treatment adequacy to reduce the functional symptomatology and burden of chronic kidney disease [1]. Over the last 20 years, erythropoiesis stimulating agents (ESA) and intravenous iron compounds have revolutionized the management of anemia in dialysis patients [2,3]. In the majority of cases, the correction of anemia is achieved easily contributing to significant improvement in the quality of life of dialysis patients, increasing physical capacity, and reducing blood transfusion requirements [4]. Anemia management in dialysis patients has been refined over time, and hemoglobin targets have been adjusted according to major interventional studies outcomes [5,6]. The most recent best practice guidelines strongly recommend partial correction of anemia and maintaining hemoglobin (Hb) concentrations in the range of 10 to 12 g/dL [7,8]. Tighter Hb targets have caused physicians to dose-adjust more frequently, with a consequent increase in Hb cycling, and there is some debate about whether or not this may lead to increased morbidity and mortality [9–13]. Preventing large hemoglobin fluctuations and frequent or prolonged excursions in a higher Hb range is now recommended by international guidelines to reduce additional cardiovascular insults [14]. Resistance to ESA action is associated with increased costs and poor outcome in dialysis patients [15]. Indeed, the cost effectiveness of anemia treatment in chronic kidney disease patients has been recently questioned [16].

Optimizing anemia management in dialysis patients has become an increasingly complex problem for nephrologists [17–20]. On one hand, correction of anemia is associated with poor outcomes when target hemoglobin levels are not achieved, while on the other hand there may be untoward effects when Hb or ESA dose are exceeded [21–27]. Hyporesponsiveness to ESA and/or its corollary (high ESA dose) have been recognized as a risk factor in hemodialysis patients [28–30]. Individualized anemia management with customized Hb targets is strongly recommended to reduce variability and potential side effects of ESA use [31]. Availability of ESAs presenting with various pharmacokinetic and pharmacodynamic profiles (long versus short acting agents, role of administration route—IV vs SC) has created an additional level of complexity in managing renal anemia [2]. Recently, a bundled payment system of anemia treatment in dialysis patients in the US has added greater complexity for care givers [32].

Recognizing the complexity of treating anemia in dialysis patients, several tools facilitating anemia management have been developed [33]. Preliminary studies have underlined the benefits of expert systems providing paper-based guided protocols and algorithms in facilitating and individualizing anemia management [34–36]. More sophisticated ESA modeling techniques using computer based decision tools accounting for personal characteristics and temporal changes in ESA sensitivity have shown potential benefits in dose adjustment [37–39]. In addition, protocol-driven management (including ESA and iron supplementation) based on electronic support has also identified staff-saving time and cost-saving potential in treating anemia in dialysis patients [40]. More recently, more refined anemia modeling using artificial neural networks has proved to be powerful and reliable tools for anemia management in dialysis patients [41–44].

The point of weakness of the previous studies on anemia management models was the limited statistical reliability for a general CKD population, due to the use of small validation test samples, often comprising tens of patients [38,41–47].

Furthermore, the key point of an anemia management model was its ability to predict accurately the hemoglobin patient level considering all the main patient features influencing anemia; this point is not deeply explored in the previous works.

The aim of this study was to develop an accurate model that in a large cohort of dialysis patients is able to predict the long-term (3 months in the future) Hb response to IV darbepoetin and iron administration as a function of patients’ characteristics. The model has been derived by means of an Artificial Neural Network (ANN) design taking into account erythrocyte dynamic and darbepoetin kinetic.

## Methods

### Study target and design

A retrospective observational study (from 2006 to 2010) on prevalent End Stage Kidney Disease (ESKD) patients undergoing hemodialysis (HD) in NephroCare clinics in Portugal was performed with the aim of developing a model able to predict the long-term Hb response to IV darbepoetin and iron therapy; an ESKD patient is considered prevalent if he/she has been in hemodialysis for at least 90 days. For this study the data were extracted from an international network clinical database (EuCliD) [48]. All patients consented in writing to the use of their anonymized data for scientific research. This observational study was not submitted to an ethics committee. No ethical approval was needed because this study was a retrospective study and purely observational (non-interventional) in nature. However, this study was revised and specifically approved by the Medical Board of Fresenius Medical Care and was conducted according to the principles expressed in the Declaration of Helsinki on anonymized data.

In the selected dialysis clinics, all dialysis parameters are recorded at each session and stored in a central database together with the results of routine laboratory analyses and periodical medical examination. This comprehensive database offers a dynamic clinical picture of patients with regular updated biochemical indicators and detailed pharmacological treatment, including dosage frequency of each medicine.

Patient inclusion criteria were as follows: patients who received at least one dose of intravenous (IV) darbepoetin alfa, and did not receive any other ESA or non-IV iron; patients having at least 6 months of follow up, that is, they received dialysis treatments in our clinics for at least 6 months; patients for whom administered darbepoetin and iron doses were in the usual ranges for dialysis patients (patients with a darbepoetin dose > 400 μg or iron dose > 300 mg were excluded as these values are deemed not reasonable). The 6-month constraint is required because 3 months of patient data before prediction date are used as model input, whereas the Hb measurement 3 months after prediction date is the target output used to train the model.

**Fig 1:**
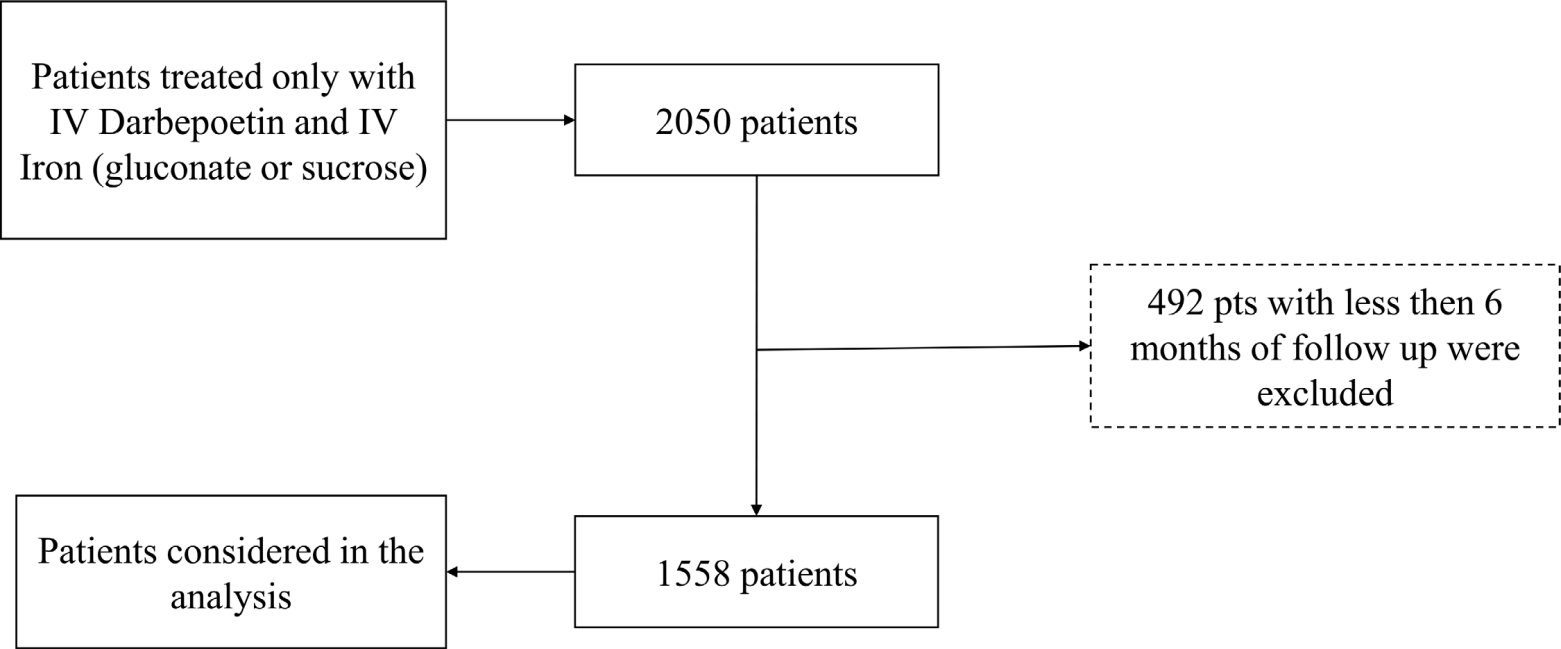
Patients’ inclusion criteria.

The initial cohort was composed of 2050 patients and after excluding patients with less than 6 months of follow up 1558 patients remained for analysis (Fig 1).

### Data collection

From the complete clinical database, we identified specific patient information known to affect individual anemia therapy response. Patients’ general information is recorded at the time of the first clinic admission and is updated periodically. Laboratory tests are performed once per month or quarterly, depending on the biomarker (Hb is performed monthly). Blood samples are extracted before the dialysis session. At each treatment, the darbepoetin alfa and iron dose administrations are recorded by the nurses, as well as usual parameters assessing dialysis performance delivery and patient clinical conditions (time, weight, blood pressure, heart rate, blood flow, dialysate flow, ionic Kt/V).

After cleaning the database and applying the exclusion criteria, 1558 patients and 35307 hematological measurements (Hb) were included in the analysis. Baseline characteristics of the studied population are presented in Table 1. Anemia management profile and Hb concentrations over the study period are shown in Table 2.

**Table 1:** Baseline patient characteristics.

Variables	Values
Patient characteristics	
Age (years)	62 ± 15
Gender (female, %)	39
Height (cm)	163 ± 8.8
Body Mass Index (kg/m^2^)	25.21 ± 4.42
Pre-dialysis weight (kg)	69 ± 13
Post-dialysis weight (kg)	67.8 ± 13
Dialysis vintage (years)	3.1 ± 4.2
Pre-dialysis systolic blood pressure (mmHg)	142.4 ± 26.5
Pre-dialysis diastolic blood pressure (mmHg)	69.7 ± 15.3
Nephropathy and causes of CKD5	
Diabetic (%)	25
Hypertension + vascular (%)	15
Chronic glomerulonephritis (%)	5
Polycystic kidney disease (%)	6
Others, miscellaneous (%)	41
Undetermined (%)	8
Comorbidities	
Diabetes (%)	32
Ischemic heart disease (%)	19
Heart failure (%)	13
Peripheral artery disease (%)	31
Stroke/cerebrovascular accident) (%)	20
Chronic respiratory diseases (%)	9
Others (%)	99
Renal replacement therapy	
*Treatment modality (LFHD/HFHD/HDF; %)	1/41/58
Vascular access type (Fistula/Graft/Catheter; %)	60/15/25
Dialysis duration time per session (min)	226 ± 14
Number of dialysis sessions per week (%)	95
**eKt/V	1.38 ± 0.39
***spKt/V	1.60 ± 0.43
Anemia therapy	
Absence of ESA (%)	13
ESA dose (μg/month)	33.7 ± 34
ESA dose (μg/kg/month)	0.52 ± 0.54
IV iron dose (mg/month)	45.2 ± 40.9
Selected laboratory values	
Hemoglobin (g/dL)	12 ± 1.5
Ferritin (μg/L)	363 ± 263
TSAT (%)	26.2 ± 16.3
Albumin (g/dL)	4.0 ± 1.5
Phosphate (mg/dL)	4.9 ± 1.5
CRP (log value; mg/L)	1.5 ± 1.49

* LFHD: low flux hemodialysis, HFHD: high flux hemodialysis, HDF: hemodialfiltration

** eKt/V: estimate Kt/V where K stands for urea clearance, t stands for treatment time, and V stands for urea volume distribution

*** spKt/V: single pool Kt/V

**Table 2:** Anemia management profile and hemoglobin concentrations.

Laboratory	Median	Range	Quartiles		
Hemoglobin (g/dL)	11.9	5.5:21.6	11.2‐11.9‐12.7		
Ferritin (μg/L)	405	7:4300	278‐405 ‐553		
**ESA and iron therapy**	**Median**	**Range**	**Quartiles**	**Administration route (IV)**	
Darbepoietin α weekly dose	20	0‐330	10‐20‐35	100%	
ERI	4.93	0‐144	2.1‐4.93‐9.1		
Iron weekly dose	33	0‐300	0‐33‐50	100%	
Hemoglobin variation at 3 months*					
**Std**	**Median**	**Range**	**Quartiles**		
1.62	0	-11.5 ‐11.7	-0.9 ‐0 ‐0.9		
**Ebben classification [14]: 10–13 g/dL (No. of 6 months Hb sequence: 27896)**					
**Constantly low**	**Constantly in**	**Constantly high**	**LAL**	**LAH**	**HA**
<1%	42%	2%	13%	35%	9%

* For each patient, Hb variation is computed as the difference between the Hb measure in a certain month and the measure performed three months after

#### Development of a predictive model for erythropoiesis based on artificial neural network and erythropoietin pharmacodynamics: theory and model assumptions

**Fig 2:**
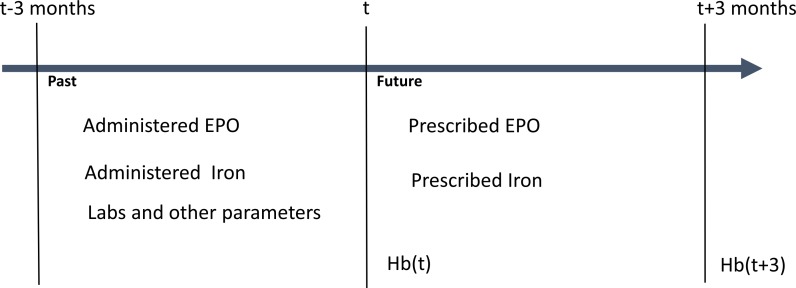
Predictive anemia modeling based on ANN. At time t the model predicts the Hb variation between time t and time t+3 months using the patient past history and the subsequent 3 months of darbepoetin and iron prescription.

The aim of this study was to develop a model able to predict long-term Hb fluctuations, i.e. an algorithm that, given the Hb concentration at time t predicts the Hb concentration at time t + 3 months (Fig 2). Predictive modeling was performed combining well-established machine learning (ML) techniques with careful feature engineering guided by the principles of actual drug kinetics to long-acting ESA therapy, specific biological dynamics and patient physiologic parameters. When given intravenously, darbepoetin alpha shows a half-life of about 24h and it remains in circulation for few days at decreasing concentrations [49]. After administration, darbepoetin alpha stimulates the RBC maturation process. Erythrocyte development requires about 13 days to be completed within the bone marrow; afterwards, immature reticulocytes are released into the bloodstream for becoming mature Hb-laden RBCs that usually survive for about 60–90 days in End Stage Renal Disease (ESRD) patients. We therefore modeled our ML algorithm considering that the present Hb levels may be influenced (with declining potency) by darbepoetin alpha doses administered during the previous 3 months [50]. This time lapse includes erythropoiesis and erythrocyte lifespan.

All the experiments were performed by means of Statistical toolbox and Neural Networks toolbox of Matlab, R2013b.

Available data were split into two dataset, one holding 60% of the records and used to train the model, while the remaining 40% of records were used to test it. Best results have been obtained by means of an Artificial Neural Network (ANN) with 2 layers of 10 neurons each [51]. ANNs are relatively flexible and use nonlinear regression tools with an architecture that is inspired by the structure of the human brain. During the training phase the ANN is presented with a collection of input-output pairs and learns, by example, to approximate the relation between such pairs; to do so, it iteratively adjusts the weights of its connections. Through this process ANNs are capable to discover and learn relationships encoded in the data and generalize them. Models are then validated on unseen examples (test phase) to assure the generalization capability. Our algorithm was designed to reproduce erythropoiesis kinetics considering that present Hb concentrations were reflecting the darbepoetin alfa doses administered IV during the previous 3 months–this time lapse includes erythropoiesis and erythrocyte lifespan [52].

Due to the different frequency of parameters sampling (e.g., Hb concentrations are measured once per month while dialysis treatment-related parameters are collected thrice per week), a merging logic combining the relevant parameters into a consistent temporal series of patient records was created. The natural choice for the timeline driver was the Hb measurement, meaning that for a given patient, a distinct record was built whenever a new Hb value was available.

**Fig 3:**
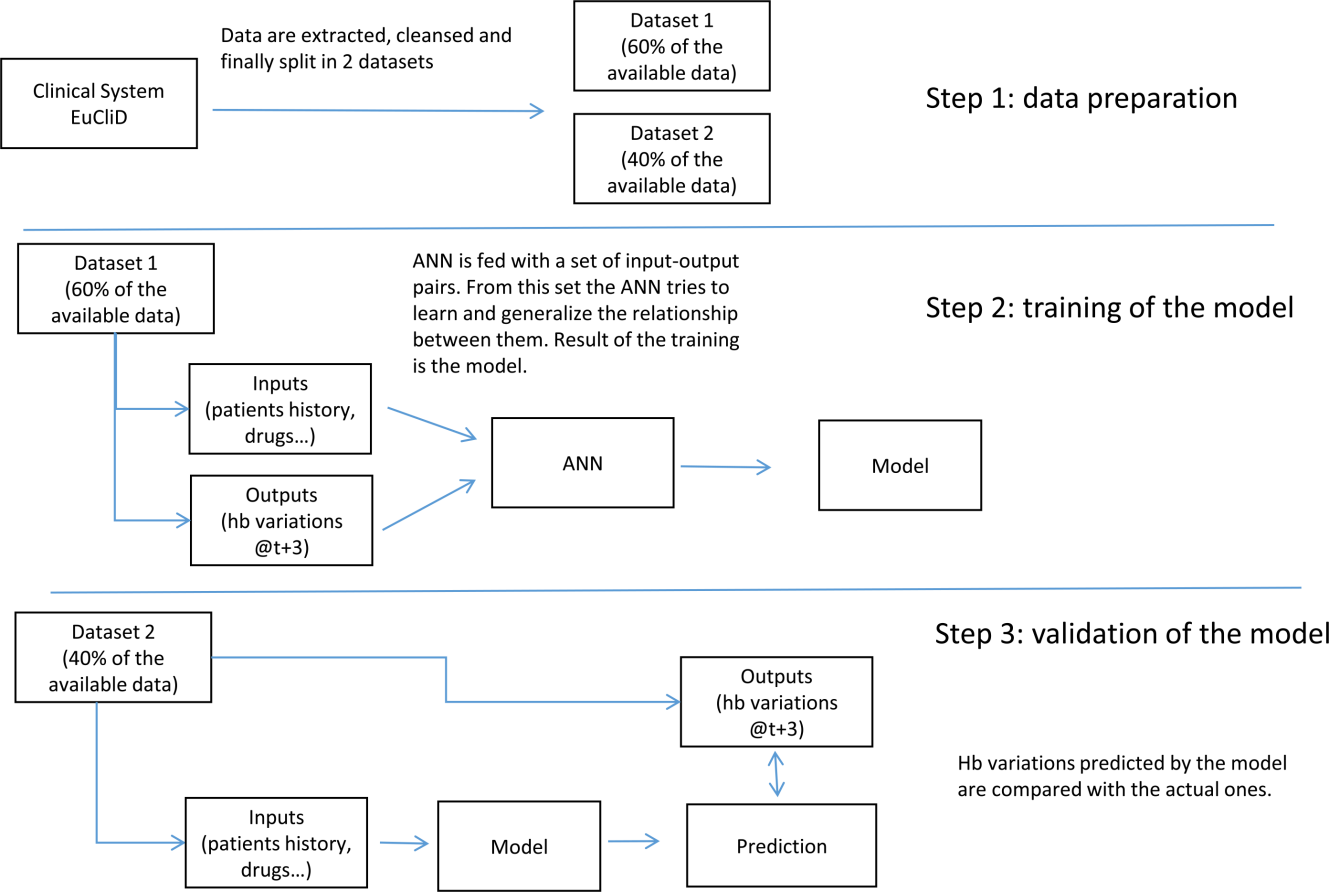
Steps involved in the development and validation of the Artificial Neural Network anemia modeling.

Darbepoetin and iron doses administered IV during the previous 3 months and prescribed in the subsequent 3 months are calculated for each Hb record. Steps involved in the development and validation of the ANN anemia modeling are summarized in Fig 3.

Additional factors contributing to Hb concentration fluctuations (e.g., inflammation markers, iron availability indicators, dialysis treatment adequacy, etc.) were included in the algorithm. Last available laboratory measurements performed during the considered period were taken, while for dialysis parameters collected at each session (e.g. Online Clearance Monitor Kt/V and weight) their mean values were calculated. The complete list of features included in the model is listed in Table 3. It is remarked that, as the study was retrospective, for each example the exact amount of darbepoetin and iron in the next 90 days is known. In real applications this information would be computed as the optimal prescription to match the target hemoglobin.

**Table 3:** List of features included in the model.

Feature
Age
Gender
Height
Pre-dialysis weight
Post-dialysis weight
Dialysis vintage
Diabetes
Treatment modality
Vascular access type
Dialysis duration time per session
Number of dialysis sessions per week
eKt/V (mean, SD)
IV ESA doses
IV iron doses
Ferritin
TSAT
Albumin
Phosphate
CRP

We also performed an analysis to identify the main causes of large prediction errors (absolute error > 1.5 g/dL) by the developed model. To this end, adverse events, defined as hospitalizations, transfusions, and intercurrent events during dialysis treatment (i.e. blood loss, hypotension episodes, and episodes of systemic infection), were isolated. For each record with a large prediction error in the training and test set, we checked whether an adverse event occurred between the time when a prediction is made (i.e. when Hb(t) is observed) and the time the prediction can be validated (i.e. when Hb(t+3) is observed).

## Results

### Accuracy of model prediction

Overall ANN anemia modeling performances in training and test phases are presented in Table 4.

**Table 4:** ANN anemia modeling performances in training and test phases.

Model Outcomes		
	Training	Test
No. of Hb measures	22859	12467
Mean Absolute Error (g/dL)	0.76	0.75
-1.5 g/dL < errors < 1.5 g/dL	88%	89%
Absolute Errors Quartiles	0.28 ‐ 0.60 ‐ 1.06	0.26 ‐ 0.59 ‐ 1.06
Errors Quartiles g/dL	-0.55 ‐ 0.06 ‐ 0.66	-0.58 ‐ 0.02 ‐ 0.59
Median Error (g/dL)	0.06	0.02
Errors Range (g/dL)	-6.5 ‐ 10	-6.5 ‐ 6.7

**Fig 4:**
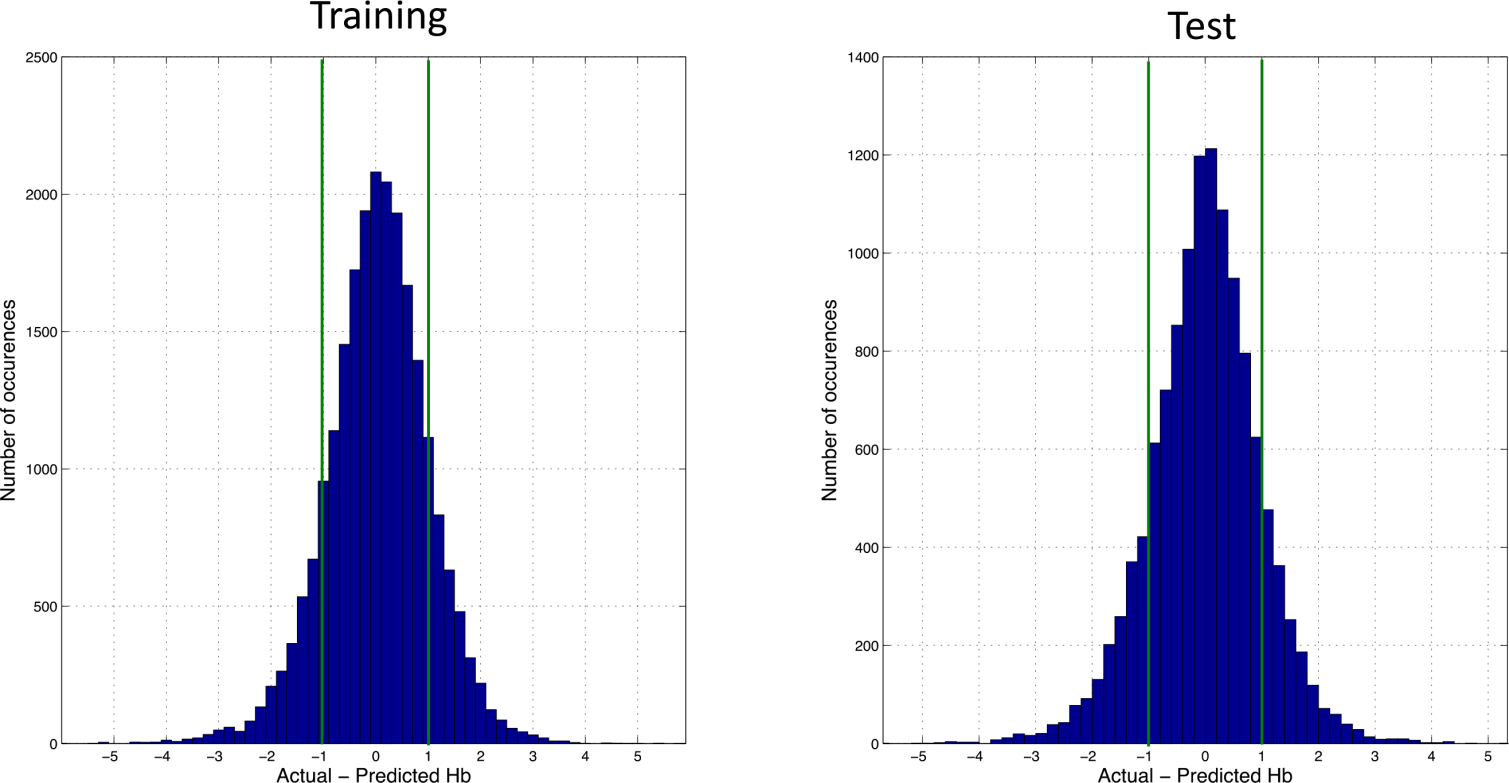
Histogram of the Hb error distribution in the training phase (left panel). Histogram of the Hb error distribution in the test phase (right panel).

The accuracy of model prediction was evaluated by analyzing the errors distribution between predicted and observed Hb concentrations during the training and test phase. This is presented in Fig 4, left and right panel respectively. A relatively narrow Gaussian error distribution centered on 0 was observed in both cases, indicating a remarkably conformity of the simulation outcome with the actual response of the population, and minimal bias in the predictions.

**Fig 5:**
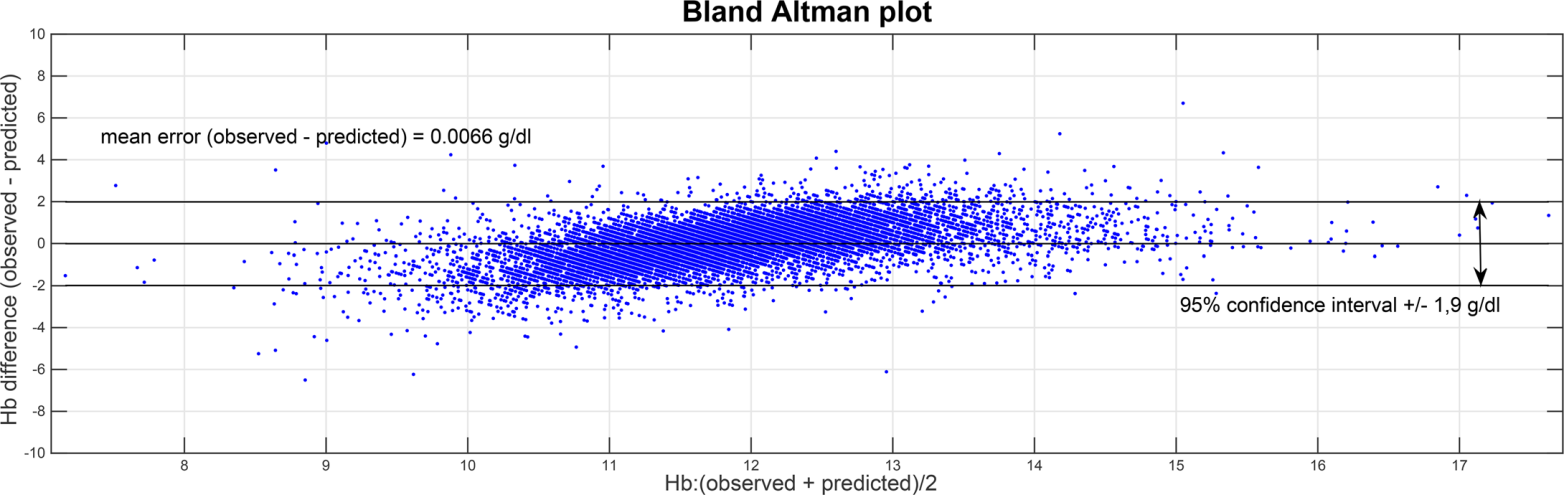
Bland-Altman analysis of observed/predicted Hb values.

The robustness of the model prediction outcomes was also confirmed by using usual metrics as shown in Table 4. Bland-Altman analysis was performed to assess the discrepancy of observed versus predicted Hb concentrations over the complete spectrum of mean Hb values of the cohort population. This analysis is reported in Fig 5. The data show a positive slope (p<0.001) due to a smoothing effect on predictions when actual Hb values tend to get to the extremes. This effect also shows when computing the mean signed prediction error for Hb quartiles (-0.98, 0.02, -0.003, and 0.94, respectively), as compared to the one measured over all (-0.007).

### Clinical accuracy and predictive value of the modeling approach: clinical cases

Two typical examples of non-conformity to the predictive model are reported here: one patient had a sudden Hb increase from 6.7 g/dL to 12.4 g/dL, and on checking this patient’s file it became apparent that he had received blood transfusions. Another patient had a sudden drop of Hb from 12.1 to 6.5 g/dL, then went back and plateaued at 13.7 g/dL over three consecutive months without any changes in anemia management or documented event, suggesting an aberrant Hb value due to laboratory or data handling error. Other causes of large errors are hospitalizations–focusing on predictions with absolute error greater than 1.5 g/dL, 1029 (642 in training dataset and 387 in test) of them occurred after documented hospitalizations, blood transfusion (102 cases) and intercurrent events during dialysis sessions (117 cases), specifically blood loss, hypotension episodes with dry weight adjustment, and episodes of systemic infection. Main causes of errors in model predictions are presented in Table 5.

**Table 5:** Main causes of large errors and discrepancies (absolute error > 1.5 g/dL) in the anemia model prediction are events that occurred in the time period of a prediction. Considered intercurrent events during the dialysis session are mainly blood loss, hypotension episodes and episodes of systemic infection.

Event	No. of predictions with |error| > 1.5 g/dL in training dataset	No. of predictions with |error| > 1.5 g/dL in test dataset
Hospitalization	642	387
Intercurrent events	105	12
Transfusion	52	50

**Fig 6:**
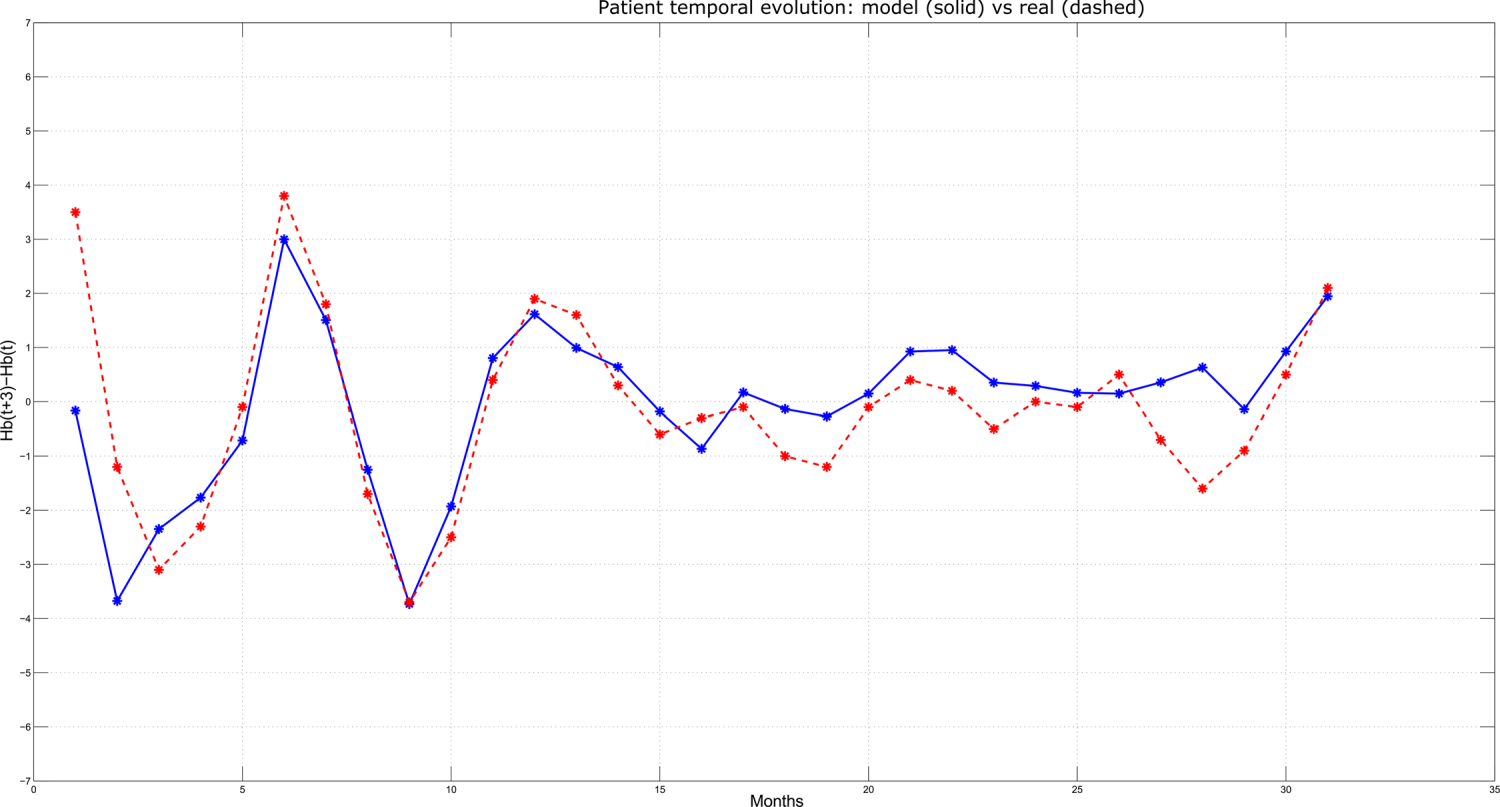
Predicted vs. Actual Hb variations for a patient characterized by a prediction error close to the mean absolute error on test set. At each time step (corresponding to monthly lab tests, on the x-axis), the Hb variation predicted by the model over the next three months (i.e., Hb(t+3)–Hb(t), on the y-axis) is plotted in solid line and compared with the actual variation observed over the same time interval (plotted in dashed line). Time steps are counted starting from the first month for which an Hb prediction for the patient was possible.

**Fig 7:**
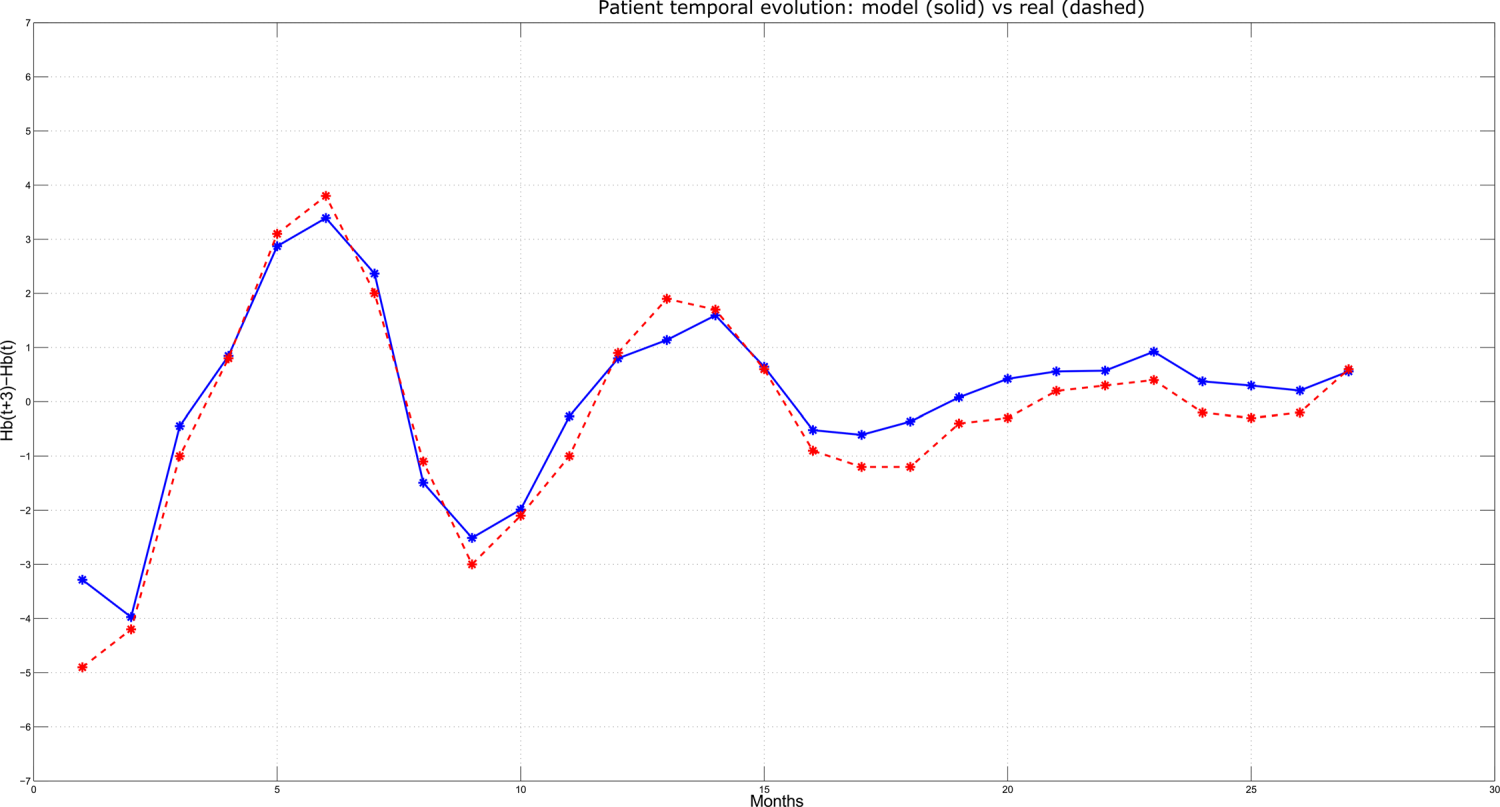
Predicted vs. Actual Hb variations for a patient characterized by low prediction error.

**Fig 8:**
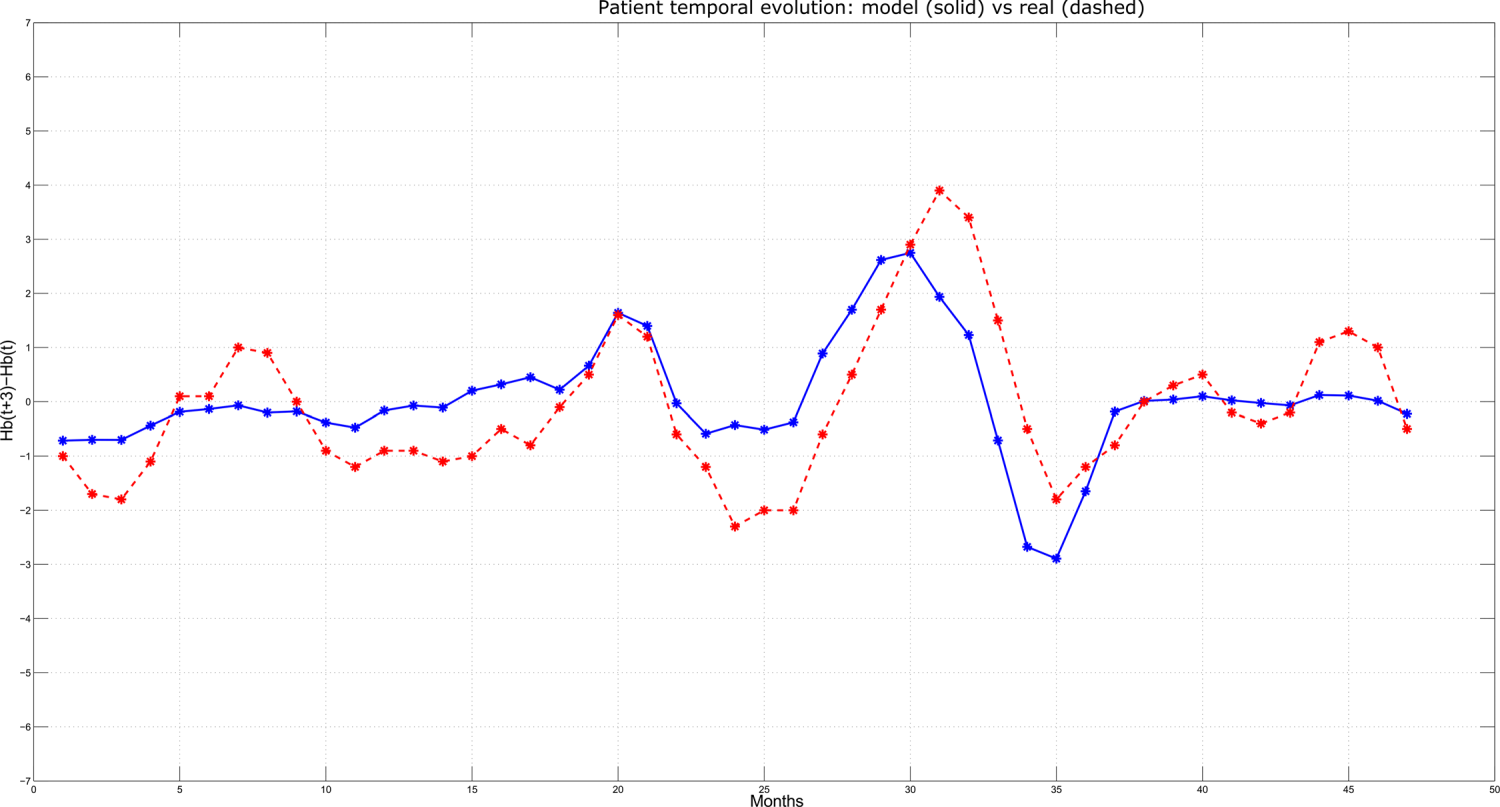
Predicted vs. Actual Hb variations for a patient characterized by high prediction error.

Among the 1558 HD patients, three have been selected and reported as typical examples of Hb concentrations behavior over time (Figs 6–8). At each time step (corresponding to monthly lab tests, on the x-axis), the Hb variation predicted by the model over the next three months (i.e., Hb(t+3)–Hb(t), on the y-axis) is plotted in solid line and compared with the actual variation observed over the same time interval (plotted in dashed line). In particular, Fig 6 reports the case of a patient for whom the model displayed an average performance, i.e. where the Mean Absolute Error (MAE) is close to the sample population mean (in this specific case, MAE = 0.74 g/dL). Simulation and real outcome display very similar trends and, although the prediction not always exactly corresponds to the actual Hb value, the model was always able to maintain the same tendency as the real data. Fig 7 describes a patient for whom the model displayed a better performance with respect to the sample population mean and very high precision in anticipating quarterly Hb variations; in fact, MAE resulted below the average (MAE = 0.45 g/dL), as clearly illustrated by the proximity of the predicted and the observed Hb values. Finally, Fig 8 shows a simulation where MAE = 0.83 g/dL, that is below the average model performance. Although the MAE is above the average, still the model is able to predict the tendency of the Hb variation over time.

To validate the clinical relevance of the predictive ANN modeling we performed analyses at patient level during both training and test phases. As shown in Table 6, average error on predicted Hb concentrations during training and test phases was less than 1 g/dL in 78 and 87% respectively. Knowing that intra-individual laboratory Hb concentration variability is ± 0.5 g/dL and that fluctuations of volemia (weight gain and weight loss) may significantly affect the precision of Hb measurements, this suggest that our ANN model provides a clinically-acceptable tool for guiding anemia management in HD patients.

**Table 6:** Predicting performances of ANN modeling at patient level.

**Patients mean absolute prediction error: Hb |measured—predicted| Training Phase**	**0 ‐ 0.5**	**0.5‐1**	**1‐1.5**	**>1.5**
No. of patients	376	794	279	57
% of patients	25%	53%	18%	4%
**Patients mean of absolute prediction error: Hb |measured—predicted| Test Phase**	**0‐0.5**	**0.5‐1**	**1‐1.5**	**>1.5**
No. of patients	990	322	88	106
% of patients	66%	21%	6%	7%

Interestingly, most largely invalid predictions (i.e. absolute error higher than 1.5 g/dL) were often caused not by the model itself but by intercurrent events or hemoglobin dosage errors occurring within the time interval of interest as described above.

## Discussion

This study as well as some previous work demonstrates that a predictive modeling approach might be a useful tool for clinical guidance in optimizing anemia management of hemodialysis patients [45]. In practical terms, the model might be used as a medical decision support tool to estimate the optimal dose of ESA/iron for achieving a desired hemoglobin variation. The main concerns with previous anemia modeling methods were the limited number of patients involved, the selection of patients, the limited available data, and the incomplete correspondence between predicted and observed Hb values [46]. In our study, the size and the diversity of the considered ESKD HD population has been essential to derive a general and reliable model for the long-term prediction of ESA/iron therapy response. Indeed, the dimension of the population under study allowed training the algorithm on different randomizations showing stable or even improved performances in the test set, which means that the model was able to well generalize the learned patterns and thus able to perform reliable prediction also on the unseen examples of the test set. Now, when compared to a similar concept reported by Gaweda et al. on a selected and restricted HD population (40–60 patients) our results compared favorably [45,47].

By using a tool that combines machine learning principles with drug kinetics and specific biological dynamics, exploiting collected patient characteristics and precise treatment features (route, dose and frequency of erythropoiesis stimulating agent and iron administration) over the prior 3 months, it has been possible to forecast Hb concentration values and Hb behavior over the next three months. Interestingly, this computational approach provides a way to reproduce individually and accurately kinetic and dynamic profiles of erythropoiesis over time in a large cohort of hemodialysis patients. The analysis indicates that the mean absolute error between predicted and observed Hb concentrations was less than 0.5 g/dL for a large part of the patients (66%). This remarkable low difference in predicted versus observed Hb values is acceptable in the clinical management of HD patients considering the usual fluctuations of Hb values due to extracellular fluid variation and laboratory uncertainties.

In the Bland-Altman analysis, a positive slope was found: this is due to the smoothing effect of the predictive model, whereby as observed Hb values get much smaller or larger than the mean (which also corresponds to more rarely observed cases), predicted values tend to get more conservative; this ensures that prediction performance is optimized over the whole population. Still, the model shows the ability to predict the direction of Hb variation also for these extreme cases–which, oftentimes, are driven by factors other than ESA administrations. Even though this smoothing effect is acknowledged, it cannot be corrected, as this would require the model to know beforehand the value of the future Hb that it is trying to predict. The Bland-Altman analysis also shows that outliers represent a small percentage of observations, again underpinning confidence in the predictive value of the model. As illustrated by some typical clinical cases, predicted Hb values and Hb behavior trend over the three month forecast were closely aligned to the actual measured Hb concentrations.

It is also interesting to note that, in cases of large or unexpected discrepancy between predicted versus observed Hb values, the root cause analysis as presented in Table 5 identified intercurrent and/or unpredictable events associated with hospitalization, blood transfusion, and laboratory error or misreported Hb values [53]. From a clinical perspective, this observation reinforces the interest for clinicians of using predictive anemia modeling in practice: firstly, it confirms that in stable hemodialysis patients, Hb concentrations follow the trend identified by the ANN model, mainly driven by the kinetics of erythropoiesis under the action of ESA and iron supplementation, while erythrocyte loss remains constant; secondly, it indicates that significant discrepancy (> 1.5 g/dL) between predicted and observed Hb values should be considered by the attending physician as a clinical warning situation (hospitalization, intercurrent events and transfusion) and not an error in the model prediction. In this case, attention of the physician is required to identify the reason (false alarm due to laboratory error, or true alarm in relation to an intercurrent event), which may require individualized management depending on the underlying diagnosis.

The strength and interest of this advanced predictive anemia modeling approach relies on several features. Firstly, it is an integrated individual approach that includes features of patient characteristics, actual anemia management, previous and individual response to anemia therapy, and temporal variations of clinical and/or biological profiles of HD patients. In this context, the reliability and accuracy of the anemia modeling is strengthened by the approximation of erythrocyte kinetic as part of the machine learning process in the previous quarterly period, which is used subsequently for predicting Hb behavior over the next quarterly period. In addition, the individual acquisition of these erythrocyte kinetic and dynamic parameters permits the development of a true and individualized approach accounting for personal sensitivity to ESA administration. Secondly, this approach provides a feedback control of anemia management in a real clinical context, which uses the actual erythrocyte kinetics produced in response to anemia therapy and probes the patient sensitivity to ESA action. By this means, it becomes possible to customize ESA dosing and iron supplementation to individual patient needs including temporal changes, without resorting to prespecified protocols relying on paper (package insert) or computer-based (investigator) decision support systems that are mainly developed for improving poor performing dialysis units or for reducing time spent in managing anemia. Thirdly, by using this approach that provides feedback control of Hb concentrations almost in real time, a more reliable and sustainable Hb target in dialysis patients can be reached more easily and quickly, at the same time reducing Hb variability over time and decreasing ESA dose [44]. All these aspects may improve the safety profile of ESA treatment in dialysis patients already prone to cardiovascular complications. Fourthly, from a health economic perspective, one might expect a cost reduction associated with an improvement of safety profile of ESA, but a specific cost analysis should be performed to evaluate the economic impact of such system.

Three months future Hb prediction based on the patient’s medical history fitted accurately with observed Hb behavior and values. In other words, our machine learning process and algorithm definition represent a good approximation of erythropoietic kinetics of a given dialysis patient; however the study intent was not to compare performances of physician prescriptions to the ANN model. Potential future application of the derived model could be to use it for physician guidance and help him in managing anemia in hemodialysis patients.

The main limitations of this predictive anemia modeling approach are twofold: firstly, the ANN analysis has been developed and tested in a retrospective cohort of hemodialysis patients; secondly, the anemia predictive model was built with only one long-acting ESA (darbepoetin alfa). Future studies of this kind should consider all ESA types. Additionally, it must be considered that the analyzed data come from a limited number of facilities acting within the same private network; thus, the model still needs to be validated on other populations.

Another limitation regards the selection of patients with at least 6 months of follow up. The model is not trained and validated on patients with less than six months of follow up in our database (lost in follow up, transfer to another dialysis provider, death…). Therefore, in the first 6 months since admission the performances and reliability of the model cannot be ensured: in real clinical practice this constraint might suggest not to use the model in this situation. Although the set of predictors used by the model covers a large portion of factors known to influence ESA response, it is necessarily not exhaustive; other variables that might affect ESA response, such as PTH [54], could not be included due to the sparse amount of available measurements.

The potential interest and future of this predictive anemia modeling approach needs to be evaluated in a dedicated and appropriately-designed prospective study. A pilot prospective study has been implemented in selected clinics of NephroCare network to assess guidance and provide value for physicians and caregivers with regards to anemia management. The study explores the accuracy of predicted Hb values as well as the physiologic profile of Hb achieved and ESA dose changes over extended periods of time. Based on this advanced ANN approach, practice pattern differences and inter-facility differences in Hb concentrations and ESA consumption may be reduced [55,56].

In summary, our predictive model using ANN offers a reliable tool for anemia management in hemodialysis patients. Based on the automated and continuous past three months acquisition of anemia treatment characteristics, patient-specific data, and laboratory parameters, ANN has the power to predict Hb concentrations 3 months into the future with high degree of accuracy. To further explore the potential value and sustainability of this ANN approach in managing anemia in hemodialysis patients, a prospective study in selected clinics of the NephroCare network is underway.
